# Antipsychotic prescribing in dementia

**DOI:** 10.1192/bjo.2021.277

**Published:** 2021-06-18

**Authors:** James McLoughlin, Emma Roemmele, Marguerite Cryan, Catherine Dolan, Geraldine McCarthy

**Affiliations:** 1 Psychiatry Department, University Hospital Galway; 2 Sligo University Hospital

## Abstract

**Aims:**

The majority of people with dementia will develop one or more behavioural or psychological symptoms of dementia (BPSD) as the illness progresses. Treating these symptoms in diverse residential environments is a challenge, with frequent prescribing of antipsychotic medications. The risks and limited benefits of antipsychotic use in this context are well recognised, prompting national guidelines in Ireland to improve prescribing patterns.

1) Assess the frequency and appropriateness of prescribing of antipsychotic medication in older adults with BPSD referred to Psychiatry of Old Age service in the West of Ireland (Sligo) by comparing with best practice guidelines.

2) Address identified deficits via quality improvement initiatives within department.

**Method:**

Audit standards were set using draft National Clinical Guidelines and NICE guidelines for prescribing in dementia to develop a study specific audit tool.

Items assessed included: the frequency of review of antipsychotic use, whether or not non-pharmacological methods were trialled, if there was an assessment of benefit of the antipsychotic and discussion or risks, if a reduction/discontinuation of antipsychotic was considered, if metabolic monitoring was achieved.

Clinical records for all patients actively under the care of the clinical team with a diagnosis of BPSD were assessed using this tool at the time of the study.

**Result:**

49 patients with BPSD were attending the service in this time period. 58% (n = 29) of the entire cohort were prescribed an antipsychotic, most commonly quetiapine. Patients cared for at home showed the lowest levels of antipsychotic use at 50% (n = 18), while those who were in nursing home (80%, n = 8) and hospital care (100%, n = 3) showed higher rates, though this sample size was too small to demonstrate statistical significance, χ^2^ = 5.12 p = 0.077.

Exploration of non pharmacological management of BPSD, documentation of discussion of risks of AP medication (metabolic, cardiovascular, falls, sedation, extrapyramidal), attempt at dose reduction or antipsychotic withdrawal were all achieved in less than 45% of cases (range 33–45%).

**Conclusion:**

This audit revealed higher than expected rates of antipsychotic prescribing in our BPSD cohort. It also revealed suboptimal documentation around the use of antipsychotics in this population during clinical interactions.

A subsequent intervention to the proforma assessment tool to prompt these discussions improved these behaviours, there was no impact on the rates of antipsychotic prescribing.

Despite increased attention regarding the limited benefits of antipsychotic medication in BPSD their use remains widespread. Due attention must be given to changing this practice in order to protect this vulnerable patient group.

